# PATHWAYS TO HEALTHY AGING IN THE UNITED STATES AND JAPAN: THE ROLE OF PSYCHOSOCIAL FACTORS

**DOI:** 10.1093/geroni/igad104.2508

**Published:** 2023-12-21

**Authors:** Ruoying Zhang, Margie Lachman

**Affiliations:** Brandeis University, Waltham, Massachusetts, United States; Brandeis University, Waltham, Massachusetts, United States

## Abstract

The dynamics of aging vary across cultures, with Japan showing a more benign picture of aging compared to the United States. While Japanese longevity and healthier older adulthood are well known, of interest is what mechanisms lead to such results. In American society, there is a strong emphasis on being independent, while Japanese society focuses on interdependence and social harmony. This study explored possible behavioral mechanisms linking these psychosocial factors to health and examined whether the pathways differ by culture. We used the second wave (2004) of the Midlife in the United States (MIDUS) (N = 1045) and the second wave (2012) of the Midlife Development in Japan (MIDJA) studies (N = 317). Participants’ ages ranged from 30 to 83. Behavioral mechanisms were operationalized as diet, smoking, and exercise. Psychosocial factors included sense of control, positive relations with others, independence, and interdependence. We computed a health measure by combining two inflammation biomarkers (IL-6 and CRP) and two cardiovascular biomarkers (systolic blood pressure and the ratio of total-to-HDL cholesterol). We expected sense of control and independence would make significant contributions to Americans’ health while positive relations with others and interdependence would be significant for Japanese’s health. The results supported the predictions, confirming different psychosocial pathways to health for the two samples, with diet and exercise as behavioral mechanisms in both cultures. The results provide new insights into the mechanisms for healthy aging in different cultures, and offer a deeper understanding of the cultural impacts on healthy behaviors and the aging process.

